# Impact of HIV on and the constructions of masculinities among HIV-positive men in South Africa: implications for secondary prevention programs

**DOI:** 10.3402/gha.v7.24631

**Published:** 2014-10-01

**Authors:** Yandisa M. Sikweyiya, Rachel Jewkes, Kristin Dunkle

**Affiliations:** 1 Gender and Health Research Unit, Medical Research Council, Pretoria, South Africa;; 2 Department of Behavioral Sciences and Health Education, Rollins School of Public Health, Emory University, Atlanta, Georgia, USA

**Keywords:** Men, HIV, masculinities, coping and adjustment, South Africa

## Abstract

**Background:**

To date, whilst there have been many published studies exploring the links between masculinity and HIV, not much work has been done to explore how an HIV-positive diagnosis impacts men's sense of masculinity and contextualizing the masculinities as fluid and changing.

**Objective:**

To explore how human immunodeficiency virus (HIV) impacts the lives of men and their constructions of masculinity through interviews with 18 men living with HIV.

**Design:**

Qualitative study involving conveniently and purposively selected black South African adult men who lived with HIV. In-depth interviews were conducted with 18 men who resided in Johannesburg and Mthatha, South Africa.

**Results:**

Our analysis suggests that the performance of risky masculinity may influence the acquisition of HIV. Yet, it also reveals that HIV can have a significant effect on men and their masculinities. Men's constructions of harmful notions of hegemonic masculinity pre-HIV diagnosis negatively affected their help-seeking behavior and coping and adjustment to living with HIV, post-diagnosis. The dominant discourse that men are strong and healthy visibly presented challenges for men when faced with an HIV-positive status. They interpreted HIV diagnosis as a loss, a sign of failure as a man, and evidence of an inability to retain control. Being sick undermined their ability to perform roles expected of them, and this led to feelings of powerlessness, worthlessness, and distress.

**Conclusions:**

Interventions with men living with HIV need to provide safe spaces for men to critically explore gender and constructions of social identities and the pressures these place on men and implications for their health. With this approach, harmful constructions of masculinities may be challenged and mitigated, and this process may render men amenable to change.

Successes with highly active antiretroviral therapy (HAART) have led to decreases in mortality and morbidity from human immunodeficiency virus (HIV), and people living with HIV are living longer and more productive lives (1–3) . However, in some quarters there are concerns that as people who live with HIV feel better, their sexual behavior may become more risky, or not change (4, 5), with the chance that they acquire resistant strains of the virus or, if viral suppression is incomplete, spread HIV (6).

In South Africa, to be eligible for HAART, adults and adolescents must have a CD4 count of ≤350 cells/mm^3^ irrespective of the WHO clinical stage (7). With the promotion of mass early HIV testing, there are increasing numbers of people who know they have HIV, but are not eligible for HAART, and it is essential that we promote safe sexual practices with this group of people as a key priority for HIV prevention in the population, as well as enabling them to stay in the health system until they are ready to start ARVs (8).

For a number of years, there has been awareness that the focus of interventions with people living with HIV should include their current psychological state including adjustment problems, depression, and a tendency to use avoidance as a coping mechanism (8). These problems elevate the risk of a person living with HIV transmitting the virus (8). However, in the general population women are much more willing to test for HIV than men and enter care with a higher CD4 count (9–11) .

To date, whilst there have been a number of published studies exploring the links between masculinity and HIV (12, 13), very little work has been done exploring how an HIV-positive diagnosis affects men's sense of masculinity and contextualizing the masculinities as fluid and changing (14).

Gender theorists, following the lead of Connell (15), have argued that ideals of masculinity are fluid and dynamic, and within a given social setting there exist multiple masculinities (see also 16, 17). Connell and Messerschmidt (18) further argue that there is constant construction and reconstruction of masculinities as the situations or circumstances in which they were initially formed change over time.

However, among these masculinities there is a shared cultural model of masculinity that is viewed as an ideal (19, 20). This has been termed hegemonic masculinity, drawing from the Gramscian notion of hegemony which implies a dominance which is attained through a social agreement, rather than through violent subjugation of others (15). The social agreement, or acquiescence, extends to those who are disadvantaged by the hegemonic masculinity (15). Within a patriarchal social context, this includes women and girls, and some alternative masculinities (notwithstanding important minorities who resist or construct themselves in opposition to hegemonic masculinity) (20, 21).

Because hegemonic masculinity operates as a cultural model it pertains within a culture (17), and there may be multiple hegemonic masculinities (especially in multi-ethic settings) (17, 18). However, within a single culture there tends to be one hegemonic masculinity and other masculinities (e.g. determined by age or sexual orientation) are subordinated or protest masculinities (22). Within a single setting, these may share important features. As a cultural model and ideal (23), hegemonic masculinity has traits that are recognized as features of ideal manhood, and these inform the practices of men (21), but they do not serve to create ‘cookie cutter’ men. In fact most men do not behave according to the ideals of hegemonic masculinity in all their practices, or all the time. For some men, they aspire to be seen as occupying a hegemonically masculine position, but may not do so either because they cannot or in some respects they choose not to do so.

In South Africa, there is a body of scholarship on hegemonic masculinity which is in agreement about certain male characteristics that are valorized. Hegemonic masculinity, rooted in patriarchy, is fundamentally based on the idea of male superiority over women (15, 21), and South African men are expected to demonstrate this and demonstrate control over women (24). Although their power over women does not stem from the use of violence, violence is accepted as a strategy for control when ‘necessary’. South African men are expected to be heterosexual and to engage in conspicuous performance of heterosexuality and male desirability, in competition with other men (25).

A South African patriarch is also expected to be head of a household and a provider, although in practice this is emphasized in the masculinity of older men (26). Men are expected to be strong and tough, and by implication healthy (12, 27). Linked to this is the idea that men are supposed to be in control of their environment. Particularly at a younger age, this may be tested and demonstrated through being able to engage in risky demonstrations of heterosexuality (e.g. multiple partners) (14), without incurring penalties, such as getting HIV which may be seen as a reflection of weakness (21, 28).

From this, it can be anticipated that having HIV may be interpreted as reflecting an inadequate (or loss of) masculinity both because it shows lack of strength and toughness, but also because it reflects a failure of being in control. Thus, men living with HIV may experience an attachment of a negative or deficit masculine identity that is meant to show that they are no longer viewed as ‘real’ man. This was paralleled by Hunter (28), writing about Zulu men, who described *isoka* as a celebrated social identity that is given to men with multiple concurrent girlfriends (29) but if they impregnated a girlfriend they were seen as disgraced men (30). In other words, they were stigmatized. Goffman's seminal work on stigma in the 1960s highlights how stigmatized social positions were resisted by those who would occupy them, either through denial of the condition that was stigmatizing, or through ‘passing’ (an attempt to conceal or get away with not disclosing invisible stigma) as not having it (31). In the context of HIV, denial may take the form of not testing in the face of illness and efforts to pass may involve rejecting opportunities for antiretroviral therapy, support groups and safer sexual practices (especially the use of condoms) (21).

Much of the work on understanding masculinities has focused on particular groups (often identified by race, socio-economic status, location in space, and time or religion) (see 32–36) , and has often resulted in the description of one masculine position in the group studied (e.g. (37).

A weakness of this analysis is that it implies that the masculinity is fixed, either through suggesting that in the population studied there is just one masculinity, or else that among the individuals studied there is only one masculinity occupied. A strength of Raewyn Connell's analysis has been the emphasis on the dynamic and multiple nature of masculinities, which gives the possibility of not just multiple masculinities pertaining in an individual setting, but also an individual man occupying multiple masculine positions at different times and in different contexts.

The purpose of the present paper is to explore how HIV impacts the lives of men and their constructions of masculinity through interviews with 18 men living with HIV in South Africa.

## Material and methods

This study was conducted in Johannesburg in the Gauteng Province and Mthatha in the Eastern Cape Province. These two towns represent two contrasting settings in South Africa, i.e. urban and rural settings, respectively.

Johannesburg is the largest city in South Africa in terms of population, and also the provincial capital of the Gauteng Province, which is the wealthiest province in South Africa. In contrast, Mthatha is a small rural town located in the Eastern Cape Province-one of the poorest provinces in South Africa.

This paper draws on 18 in-depth interviews conducted with adult men living with HIV. All participants were black African men between the ages 18–49, and all but one identified themselves as heterosexual. Eleven men resided in Johannesburg central or in Soweto, a nearby township. The other seven men resided in Mthatha and surrounding communities.

Interviews were conducted between 2008 and 2010 by two male interviewers. Convenience and purposive sampling techniques were employed to recruit the men into the study (38, 39). In Johannesburg, we employed convenience sampling to recruit men who were attending two local clinics as out-patients, one in town and the other in Soweto. These clinics had a dedicated service for men living with HIV. The majority of the interviews in Johannesburg were conducted by a male research assistant who was in his late 20s and experienced in conducting qualitative interviews. Prior to conducting interviews, he was trained on research ethics and techniques of conducting in-depth interviews on sensitive topics. Whilst he worked and lived in Johannesburg, he did not know the men he interviewed prior to interviews. YS, who is an author is this paper, also conducted three interviews in Johannesburg, and all seven interviews in Mthatha. To recruit participants in Mthatha, we employed a purposive sampling technique. YS contacted a male colleague who worked in the HIV and health sector in Mthatha and requested him to assist in recruiting men who lived with HIV that he knew or had worked with in his health promotion programs.

There was no predetermined number of interviews to be conducted, rather we interviewed until saturation, i.e. when no new information was emerging in each question of interest to the study (39). Each participant was interviewed once. Researchers may have preconceptions about the study topic, and this may influence the data collected and its analysis. To reduce this potential bias, we used the technique of bracketing where we attempted to put aside what we thought we knew about the topic and remained curious and open to the information we were getting in the interviews and findings emerging from the data (38). Interviews in Johannesburg were a mixture of IsiZulu, SeSotho, and English, and interviews in Mthatha were conducted in IsiXhosa, the dominant language in the Eastern Cape Province.

The scope of enquiry was developed to guide the interviews. It included asking men to share their views and experiences on how being diagnosed with HIV had affected their lives, and how the diagnosis had made them to view themselves as men. We asked men to share life stories on how they have experienced living with HIV and how living with HIV had evolved over time, if it has. Men's sexual self-expression including sexual risk taking and protection was also explored for both the pre- and post-HIV diagnosis periods. We also explored whether men acted or behaved differently as men who lived with HIV when in public spaces as compared to when in private spaces, and whether was there a difference between pre- and post-diagnosis periods. However, new issues that were not initially in the scope of inquiry but had emerged during the course of the interview and were relevant to the study topic were explored in the interview, and then incorporated into the scope of enquiry for further exploration in subsequent interviews with other men. All interviews were audio-recorded.

### Data analysis

Audio-recordings of the interviews were transcribed verbatim and translated into English in preparation for data analysis. Data were analyzed inductively employing thematic content analysis. However, there were deductive elements to the analysis as YS who led the data analysis reviewed the literature to explore themes that had been presented in similar studies and thereafter checked for those in our data (38–40) .

YS first read the transcripts repeatedly to familiarize himself with the content of the transcripts (40). Thereafter, he established few broad codes which somewhat corresponded with the questions in the scope of enquiry (39). Thereafter, text which seemed to fit together was grouped under a specific code. Subsequent to this, he explored the data and identified numerous open codes. Further to this, similar and or related open codes were grouped together under clearly defined themes. All authors then explored the relationships between the themes and interpreted what they saw emerging, but also compared the findings with those of other studies and drew the conclusions presented in this paper.

### Participants and socio-economic circumstances

Participants gave varying reasons why they tested for HIV. Twelve men said they tested because they had been sick and a HIV test was recommended either by the treating doctor or nurse or family member. Four men said they tested following their intimate partners’ death which often was suspected to be AIDS related. Three men reported that they were encouraged by their partners to test, with some of the partners having first disclosed to the men to be living with HIV. One man said he tested for HIV as a requirement to participate in contact sport. At the time of the interviews, the period men had lived with HIV ranged from being recently diagnosed (approximately 2 weeks) to 8 years.

In terms of men's employment status, at the time of the interviews, three said they were working in the HIV field as lay counselors or doing other HIV voluntary work. Two men were self-employed, four said they were formally employed doing office-based work, while eight men said they were not working. We do not have information about one man's employment status. Furthermore, we did not collect information on the education level of the participants.

We asked men about intimate partners and 14 men said they were currently dating or had a sexual partner, and the number of sexual partners ranged from having one to four concurrent sexual partners, few men reported to be currently in a relationship with a woman who was living with HIV. Sixteen men reported that they had disclosed their HIV status to at least one person. However, two men said no one knew about their status.

### Ethical considerations

Ethics approval was given by the ethics committees of the University of the Witwatersrand, South Africa and Emory University, United States of America. The purpose of the study, procedures involved, risks and benefits, and informants’ rights were explained to the participants before they signed informed consent. A total of R50 (US $5.7) was given to the informants for taking part in this research. Data presented in this article were anonymously processed with names of all the informants replaced by pseudonyms and potential identifies removed. We envisaged that some men may have strong emotional reactions to some of the issues that will be discussed in the interviews. As such, we prepared study information pamphlets with a list of local services that men could contact for psychological assistance should they feel the need. However, none of the men requested or took up the offer for referral.

## Results

In the following section, we present a synopsis of the main results. We show that HIV testing can set in chain a set of experiences that result in radical change to a person's self-assessment of masculinity as well as public assessment, depending on experience of care seeking, ill health, disclosure (voluntary or involuntary) and reactions of others to this. Thus, presenting a situation in which masculinity appears at its most fluid, with multiple masculine positions (emphasized masculinity, traditional masculinity, and diminished masculinity) presented as being occupied or aspired to over time, and existing at one time within the imagination and practices of individual men. Additionally, we show that the context of change provides the possibility of reshaping of masculinity to construct masculinities (resourceful–responsible men) based on ideas of caring and protecting which are less sexually risky and enable treatment adherence. Furthermore, we illustrate how shifts in identity can have an enduring and critical impact on men's ability to access and adhere to care and safer sex practices, and highlight the possibilities this creates for intervention to promote easier adjustment to a HIV-positive status among men.

### Emphasized masculinity

Men's practices and masculine identities before they acquired HIV were only visible through their discourse of their practices and priorities around these in the interviews we conducted with them when they had already acquired HIV. In our analysis, we noted fluidity, and sometimes blending, of masculine positions within individual man. This fluidity seemed to depend on various factors including time, space occupied by men, and, through historical accounts, age and showed that these masculine positions are not fixed.

The accounts highlight many of the features which were mentioned above as being indicative of a dominant masculinity, especially among younger people where heterosexual performance is greatly emphasized. This is illustrated in the narrative below:

Well I have started using condoms now, I wasn't practicing safe sex and I had many girlfriends and me and my friends were competing about having many sexual partners though I was from a poor family but that didn't affect my performance in having many partners. (Menzi)

Menzi was one of the 11 men in the study who indicated that he had previously sought to occupy a position that would enable him to be recognized as a ‘real’ man. He describes competing with other men on his sexual performance, measured in terms of having more girlfriends. The competition with other men over girlfriends echoes that described by Wood and Jewkes (37) among young men in the rural Eastern Cape.

Men indicated that they were socialized to understand that to be a man they should take risks, be brave and demonstrate strength, have the ability to propose love to women, initiate and sustain relationships with multiple women; be dominant and in control in such relationships.

In this context, the performance of the emphasized masculinity seems to be deeply grounded on the socialization of these men. Luxolo further illustrates this when he explained:

And we were raised in a manner that we are men, you find that we behave in a manner that we like and we socialize [party] … do you understand? And as men we have to smoke and have the drink [alcohol] do you understand? To sleep [have sex] wherever and whenever and with whomever you meet, do you understand? You are a man. (Luxolo)

Whilst it may seem that Luxolo was generalizing, and perhaps exaggerating, he was also reflecting social norms of male behavior. It is not clear whether he was talking about himself here, but he suggests that he was, and other men in this study reported similar pressure and behavior in the interviews.

In the analysis we also noted that some of the men were clearly still identifying with the youthful masculinity and at the time of the interview had several sexual partners.

### Traditional masculinity

In this setting, the traditional masculinity position is considered a mature masculinity (30). This masculinity emphasizes the expectation that men should provide a secure home for the family, fulfill a role as providers, and ensure the survival of the family line through childbearing and raising of children (41).

In our analysis, there appeared to be a blurring between the emphasized and traditional masculine positions in this setting. However, we also noted a difference between these masculine positions in terms of views on what is successful manhood and practices associated with these. The main area of difference was that the emphasized masculine position is more a youth masculinity; in contrast the traditional masculinity position is a more mature position that is tightly correlated with particular expectations and responsibilities fitting of a man who has transited from being a youth to attaining the status of adulthood.

In our study some of the men were in a different life stage. Itumeleng and Jeff were cohabiting or married, and Thabo was divorced. Itumeleng stayed with his partner and a child in a flat in Johannesburg and was the main provider in the family. Jeff was married, and had three children.

Other men, faced with the loss of their youthful masculinity after HIV diagnosis, grieved. The interviews suggested that in so doing, they mostly did not hanker after the youthful position that they had occupied and lost, but rather lamented an inability to occupy the more traditional masculinity of Jeff and Itumeleng, the one hegemonic in this setting. In other words, they grieved the future they felt they could not attain and not the past that they had lost. Tshepo described his loss in terms of an inability to occupy the carefree hegemonic masculinity, i.e. the traditional position:

You know I like women, so for my situation now I can't have many of them and I should use protection every time and the other thing is that I can't have another child because of my status.

Jeff and Itumeleng were among the men who grieved a loss of traditional masculinity. They appeared to grieve an inability to occupy a masculine position which ostensibly it appeared they were already in. For these men, it appeared they were framing HIV as a loss and judging what is lost in terms of a traditional masculine position. In this respect, they were similar to many of the men who were still in a more youthful masculinity.

In the interviews, there was a great deal of angst among men when discussing issues of childbearing and fatherhood. Men who had no biological children showed strong emotions of sadness and regret when discussing this topic. For them, having HIV meant that they would not leave anyone behind to perpetuate their family name, proving them to be failures in the eyes of their families. The following narratives illustrate this anxiety among some men.

In my life I planned to have children but after finding out about my status I just knew that I won't have children because of my status. (Joel)

Eh indeed it does not make me to feel good for sex since I'm a man, I need to revive the family name so I cannot do that because I always do it with a condom … there is no choice that I can make because I will make a poor soul dirty (transmit HIV), it is better that I just …, use a condom. (Katlego)

In contrast, men who already had children worried about the potential inability to provide for them; about dying prematurely and leaving their children without a father to provide for them; and others recognized with sadness that they will not be able to have other children. For example, Jeff stated:

That one [having children], I dream about, … I tell myself as long as I've got two kids there's no more kids, there's nothing I can do I'll never have another kid.

Luxolo talked about his wish to live longer so that he could raise his children:

I wish to find myself staying in a house or next to my place or next to the kraal you see then spending my pension money … being that man who has extensive experience in life where they [children] keep coming in and out saying I have a certain problem daddy and share tirelessly with my children … that is the thing that scares me, that seems difficult for us as men in this HIV/AIDS issue. (Luxolo)

As we note in the extract below, being sick undermined men's ability to perform their roles and led to feelings of powerlessness, worthlessness, and distress:

So then I am sitting here on the bed just as the month is coming to an end I don't know how I will pay the rent, what are the kids going to eat, I wish to be well. (Luxolo)

Providing support to this notion of manhood, Katlego highlighted the importance for him, as a man, to find a job, work and provide for his family. He explained:

So it's like I am saying that I am washing cars in town at the rank. I am able to support my family with that money. (Katlego)

In this sample of men, the traditional masculine identity is largely an aspired one and appears to most be present as a preoccupation post-HIV diagnosis.

### Diminished masculinity

HIV compromised men's ability to sustain the traditional masculine position – one that is hegemonic in this setting, and in response, many of them found themselves forced into a deficit identity. That is one that was at odds with the traditional notions of masculinity they so aspired for (42). To date, there has been little emphasis in scholarship on the emotional vulnerability of South African men, with some notable exceptions (e.g. (23). In our analysis, the diminished masculine position was particularly characterized by feelings of helplessness, hopelessness, and men's interpretation of their lives as compromised or constrained by having acquired HIV. Nine men occupied this position after they were diagnosed with HIV.

There was some distinction among men who reported this. Men who had been recently diagnosed (approximately within 1 year), at the time of the interviews, were more likely to report the emotions mentioned above. Yet, men who had lived with HIV for more than a year were more likely to mention this position in their past. The latter group had over the years, departed from this position as they adjusted to their diagnosis (see also 10, 43). For example, Itumeleng who had been living with HIV for a prolonged period of time reflected:

After I tested positive I felt that something very important had been taken from me by somebody you see and that thing I will never get it again, so I felt very weak and I felt that I'm powerless, I'm powerless you know what I mean and ja you feel like that once you have acquired HIV, you become stuck and helpless you know and hopeless.

Feelings of dejection after diagnosis were reported by other men as well, best illustrated in Thabo's narrative below:

At first when I learned that I had acquired HIV, it wasn't easy for me and I used to lock myself in the bedroom especially when it was time for me to take vitamins, but my family would tell me that it is not the end of the world.

Several other men reported feeling diminished by having acquired HIV. In their narratives, there was a great sense of loss:

Things that affect men is losing dignity, self-esteem because as a man you are a leader but if you are living with HIV it is difficult to exercise that power … it can also affect your relationship with your partner … and as a father you have to be supportive to your family and how are you going to do that when you are the one who needs support. (Menzi)

A man looks and looks and realizes that this thing that he has involved himself in [acquiring HIV] makes his life cold. (Luxolo)

The practices that some men identified as vital and marking successful manhood included strength and bravery. As such, for them, having acquired HIV and being sick, being cared for by others in the family or community, or seeking help were signs of weakness which undermined their manhood.

You know when you get sick it's a problem because many people would want to know and if you tell them they will start to feel pity for you and it's something that men don't want … and culture really, men believe that clinics are for women and if a man goes to the clinic it means that he is weak. (Menzi)

Menzi clearly read his HIV status and its impact on his health in terms of his masculinity. It was unmasculine to be ill and pitied, and to attend a clinic to seek care. HIV represented weakness, and that was unmasculine (c.f. (44). This resentment of illness could also reflect an avoidance of the reality of living with HIV and a negative coping strategy.

Some men said that the HIV-positive diagnosis necessitated behavior change in their lives. Yet, for many, making such changes seem not to be out of their own volition, rather the changes ran counter to how they preferred to position themselves as a man. It seems changing their behavior was a burdensome process for them as Luxolo explained:

That indeed in the morning and evening I will take this thing [ARVs] and for the rest of my life I will never do anything else so they are just things that you see that we are totally forced into that you must do a certain thing, do not do a certain thing you see. (Luxolo)

It appears Luxolo felt that he was living a prescribed life, and that he had lost the freedom to live his life the way he desired. His lack of control over his own life again reflected a lack of access to the hegemonic masculinity.

For other men, the need to reduce sexual partners, and to use a condom consistently when having sex were some of the changes men felt were forced upon them because of their status. This is evidenced by Boitumelo's assertion that ‘using a condom is not easy sometimes’.

What is depicted in the above narratives as an occupied position by these men is diminished manhood, a loss position that was fairly consistently described as following the diagnosis.

### Coping mechanisms: avoidance or acceptance

The men interviewed spoke of the impact of HIV in diminishing their manhood as being intolerable. Thus, they sought ways of shaking off their feelings of inadequacy which in an immediate sense stemmed from their perception of diminished masculinity but this itself was a response to being told they had acquired HIV. The data suggest that there could be three ways of doing this. The first was through deceiving partners and using traditional healers and treatments rather than clinics. As shown in Table 1, many men only tested for HIV when they were sick, and some only sought medical help at the behest of their families, or on recommendation by the doctor or nurse consulted. Men in our study, such as Menzi above, described public health centers as spaces they were uncomfortable to access and resisted doing so.

**Table 1:** Sketches of research participants

Name	Relationship	Social position	Year diagnosed	Support group attendance	Province	Why tested	Date of interview	Disclosed
Luxolo	Dating	HIV volunteer work	2000	Yes	EC	Required for participating in sport	2009	Family/publicly/partners
Ntsikelelo	Casual sex partners	Self-employed	2002	Yes	EC	Was sick	2009	Mother/friends
Itumeleng	One partner	HIV counselor	1993	Yes	Gauteng	Seems was sick? Developed glands 2 days after diagnosed	05/2009	Family/church/partner/publicly
Jeff	Wife and three girlfriends	Working	2009	No	Gauteng	Was sick and doctor recommended testing	05/2009	No one
Tobias	One partner	Working	2009		Gauteng	Advised by girlfriend	05/2009	Younger brother
Katlego	One partner	Self-employed	2003	Yes	Gauteng	Was sick	05/2009	Family/friends/clients
Mbongeni	One partner	Working	2005	No	EC	Encouraged by partner (HIV +) to test. Previous partners had died	07/10/08	Partner/family/friends
Menzi	Four sexual partners	Working	2000	No	Gauteng	Was sick	07/10/08	Family/friends/partners (All partners HIV+)
Thabo	Divorcee and one partner	Not working	2008	Yes	EC	Was sick	12/11/2008	Family
Lebohang	No partner	Not working	2008	Yes	Gauteng	Was sick	13/11/08	Family/friends and neighbors
Joel	Two partners	Working in the HIV field	1995	Yes	EC	Was sick and partner died	14/11/2008	Significant others/family/partners
Tshepo	One partner	Not working	March 2008	Yes	Gauteng	Partner had died	15/11/08	Brother
Oupa	One partner	Not working	2005	No	Gauteng	Was sick and had TB	20/11/2008	Family/ partner and neighbors
Boitumelo	Two partners	Not working	2004		Gauteng	Was sick	03/12/2008	Family/friend and one partner
Johnson	Two sexual partners	Not working	March 2006	No	EC	Was sick and in prison	10/12/2008	Mother
Mthuthuzeli	Girlfriend (HIV+)	Not working	Late 2006	No	EC	Was sick	11/12/2008	Family/ friends/ ex-girlfriends and current girlfriend
Bophelo	No partner	_	12/2008	No	Gauteng	Partner suspected of having HIV	12/2008	No one
Benjamin	Abstaining and not dating since partner's passing	Not working	May 2008	No	Gauteng	Partner had died	03/12/2008	Family/friends

This type of denial was seen again in the men who did not disclose their status to their intimate and or sexual partners as they feared a negative reaction. They feared being blamed for placing their partners at risk of HIV, although ironically were continuing to do this by non-disclosure. Additionally, they feared that their acquisition of HIV may be linked to promiscuous behavior on their part. Some said this link between men's promiscuous behavior and HIV acquisition often lead to blame and stigmatization by others in their communities. Ntsikelelo's comment to follow is illustrative:

Ey some men are boisterous others are fearful they [partners] will say they acquired the virus maybe through bad behavior … most men think that they are going to break up with the person that they were dating, they are going to break up with their wives if they ever explain that they have the virus. (Ntsikelelo)

Earlier we argued that, for some men, having many sexual partners was an essential part of being a man, so in this context this appears paradoxical. The paradox can be explained through understanding that it is the failure to be able to have lots of partners and stay in control of the situation that is perceived as unmanly, in this case loss of control is demonstrated through having acquired HIV. Our data show that these men were aware of the importance of disclosing their status to their partners, yet they thought that disclosing their status was a huge risk for themselves, their relationships and families, and they were, at least initially, not prepared to do this.

This risk perception may have had obtained credence from the lived experiences of some of the men who said that after disclosing to their partners, they experienced severe and harsh backlash. They reported being blamed for transmitting the virus to the partners, ridiculed, and called names, and for some, their wives or girlfriends left them. For instance, when Luxolo disclosed his status publicly, he experienced hostile reaction from some of his former girlfriends and the community at large. He said:

I disclosed to the community over the radio … It was very hard I was ‘that’ person, I realized that I had my ex-girlfriends that I dated when I was still young and knowing that even then the virus was not existing then, I was being followed, insulted and I understood others were going to open cases [press criminal charges]. (Luxolo)

Assessing the risk as huge, some men concealed their status from their partners. This decision, however, had negative health consequences for men and their partners. Some men said they found it difficult to take their medication in the presence of their partners and were forced to hide their medication resulting in treatment non-adherence. Other men said they continued to have unprotected sex with their girlfriends or wives even though they knew they were potentially transmitting the virus to them.

Using traditional healers seems to have had been a preferred option by some of the men. When we interviewed Bophelo, he had recently been diagnosed with HIV and mentioned having asked someone to prepare traditional medicine for him to cure the HIV:

Interviewer: Can you tell me who have you told about your status?

Bophelo: No one, I only found out this week but I just asked someone to prepare me muti (traditional medicine) that heals HIV.

Interviewer: does that person have information about HIV?

Bophelo: Yes, he had AIDS, his wife died of AIDS and when he tested he was told he has got AIDS. So when he was at church he had a vision of muti that cures HIV. He drank the muti and when he went back to test he was told he was HIV negative. So he is committed to help others … I will take it (ARVs) but I will not stop taking the muti treatment because I know muti has helped many people.

Highlighting perhaps another reason for preferring *muti* as a treatment for HIV, Itumeleng located its use as an HIV treatment to cultural practices. He explained:

African [black] men wouldn't mind to drink imbiza [traditional medicine], to induce vomiting and do all these traditional rituals and don't bother about using the condom because they want to prove that that culture is more important to them than any other thing.

Notwithstanding public education around there being no HIV cure, some men consulted traditional healers because they still held out a hope for being cured. Others preferred traditional healers as they hoped that the latter would explain away their sickness in terms other than having acquired HIV.

Another avenue of avoidance of diminished masculinity was in alcohol and drug abuse (see also 46). Stanton and colleagues argue that coping through avoidance may predict maladjustment and lead to distress for those using it (45). In our study, while some men may have also been using these substances prior to HIV diagnosis, it seems for others, post-diagnosis substance abuse may have been a coping mechanism. For instance, some men reported that they drank heavily as ‘a way of dealing with the diagnosis’, ‘to relieve the pain accompanying diagnosis’, and ‘to make their lives bearable’. This is best captured in Jeff's interview to follow. At the time of his interview, he had recently been diagnosed and was visibly emotional and teary in the interview. He narrated how he felt and how he used alcohol as a way of dealing with the diagnosis, a coping strategy. He explained:

That's what I was trying to do by the time I was home, I couldn't eat, I couldn't sleep, but … I tried too much booze [alcohol], I'd say ‘hayi [no] this is crap’ … I can't sleep while I'm drunk, it's a waste of money, waste of time, waste of everything, you just go back there, do whatever.

Consistent with Jeff's experience, Luxolo highlighted the common use of excessive alcohol as a way of avoiding to confront the reality of the diagnosis. He posited:

Another person will tell himself that it's something that is waiting for me, I am going, as I think I am waiting for death, and some of them [men living with HIV] say I am leaving work, another person may end up telling himself that he will succumb to alcohol to try and dismiss this problem which he has in his head, if I am always drunk it seems I might feel okay.

The third coping mechanism resulted in a state of empowerment through acceptance of one's HIV-positive status. Almost all men interviewed spoke of the importance of accepting one's HIV status, but this is not to say that all of them had concluded this stage of acceptance. Some were clearly still battling to accept the HIV diagnosis. Notwithstanding this, our data suggest that reaching a stage of acceptance and disclosure of their status (sometimes publicly), facilitated the process of help-seeking and treatment adherence. Whilst there were men who had negative experiences of disclosure (e.g. stigmatization), there were those who said they received support from the people they disclosed to even if those had been hostile initially (c.f. 46).

Being accepted and supported by significant others was valued by and crucial in empowering men to move from the diminished masculine position and reconfigure another masculine identity which allowed them to live positively with HIV. The following narratives are illustrative:

Just living with HIV, one becomes depressed and wants to be alone, it takes time to adjust and accept until you reach a point where you need to choose the life that you want. (Lebohang)

Ja, I even go to church, church are the ones who will tell you that there is life even if you have got AIDS. If you go there those people are so supportive there, praying for you sometimes you can be …, I can even feel that I can live with those people who ever is there. (Jeff)

### Resourceful–responsible men

Post-diagnosis, nine men positively reconfigured their masculinities and repositioned themselves as leaders, HIV activists and advocates, and were viewed as resourceful persons in their communities (see also 47). In our analysis, we noted particular characteristics among these men. They were mainly those who had lived with the HIV for a prolonged period of time; were more likely to have been through extensive HIV counseling and education or had been part of a local support group for an extended period of time; had disclosed their status to their families, intimate partners, and community; had disclosed publicly about living with HIV.

Whilst most of these men had no formal training on HIV counseling and life skills, most had taken up the role of being HIV peer counselors and performed such roles principally drawing from their experience of living with HIV over a lengthy period of time.

In their narratives, there was a relatively strong desire to make the lives of other people living with HIV bearable or to protect others from acquiring HIV. In his interview, Luxolo spoke at length about the role he was playing to support other people living with HIV in his community. It seems he was motivated by the need to prevent emotional and physical pain and suffering that could potentially be experienced by people living with HIV, if there was no support. He elucidated:

Those who have just tested because I know the challenges and that they can …, so that they do not experience them the same way I did since I didn't have any support I didn't know anything. (Luxolo)

Mthuthuzeli and Ntsikelelo also reported that they encouraged other men who lived with HIV to seek help and adhere to their medication. They motivated these men through referring to their life stories of living with HIV, as examples:

I encouraged some guy at the clinic about taking treatment, he didn't believe that our medication will help him and I had to tell him about where I come from. I told him that taking medication helped me a lot. (Mthuthuzeli)

In his interview, Ntsikelelo narrated a story where he actively played a caring role where a man in his community was refusing help and not taking his medication:

I was looking after him, he was a person then who used to not attend the clinic even when he was sick no he was not attending the clinic, he used to be a person who will sit and not even take the tablets … but when he fell sick I made him to meet the social workers then so that the caregivers at the hospice … I said to them then they must look after him. (Ntsikelelo)

Boitumelo spoke about the role he was playing in his community to prevent people from acquiring HIV. He motivated them to use condoms consistently when having sex, and echoed the same sentiment that he did this to prevent other people from experiencing the suffering he did after he was diagnosed with HIV. He posited:

I tell them about condoms and also give them condoms to use because I don't want them to suffer the way I have suffered. (Boitumelo)

In some cases, men were sometimes asked by local families to talk with a family member who was suspected of being sick with an AIDS related disease, but was refusing to seek help or take medication.

Okay, my family and friends and even in the community when someone is ill they would call me but sometimes they would call me for a person who is about to die. (Joel)

These men derived a sense of importance and self-worth from occupying these caring positions in their communities, positions that are certainly counter to hegemonic ideals of masculinity. In the following extracts, we note the feeling of self-worth which, in turn, seems to have helped these men to regain self-respect and aided them in reconfiguring their masculinities into positive ones.

When they keep talking about the HIV/AIDS issue I sometimes disassociate myself from it, I'm always on this side where I am expected to help when they say there is a person with a problem, I ask myself who is helping them, how can I reach that person and be able to assist him? (Luxolo)

There's nothing like this that I am going to tell now that has made me happy in my life, it makes me feel good especially when I'm out with guys standing in front of young girls and boys 13 years old and sharing ideas with them telling me what they know especially about sex and sexuality and me sharing ja my experiences in life like in terms of sex and sexuality and leaving them with that question mark of deciding what to do, because such thing is taboo. (Itumeleng)

By protecting their sexual partners from acquiring HIV from them by consistently wearing condoms when having sex; abstaining from sex; reducing the number of sexual partners; these men viewed themselves as responsible man and protectors. These practices are often used to describe a mature and responsible man in this setting. Thus, on noting the role they were playing in their communities, this may have restored their sense of manhood and perhaps facilitated rationalization about being worthy men in a different way. The following narrative from Katlego supports our interpretation:

Katlego: So in order for me to come to that decision, I thought of that first, then I made a decision that I will always be a person that I will have sex with I will need to have a condom

Interviewer … how does that make you feel on the inside my brother?

Katlego: Eh it makes me very happy instead of killing the community … to protect another person.

## Discussion

In this paper, we have argued that the men interviewed in many ways read their HIV diagnosis and illness in terms of their masculinity and its impact on it. Our analysis revealed that some men described having occupied the emphasized masculine position which we argue is a youthful masculinity, even though some older men may also occupy this position, that is characterized by pleasure seeking and having multiple concurrent sexual partners amongst other things (see also 29, 48). Most men described having occupied this position mainly before they were diagnosed with HIV, with most reporting having slightly departed from this position as they felt they could no longer occupy it after acquiring HIV. Reasons given for the departure from this position are consistent with Hunter's finding on *Isoka lamanyala* (a dirty and unacceptable masculinity) (30) which he described as a negative labeling given to Zulu men who had, according to the dominant culture of the time, ‘gone too far’ in their enactment of masculinity. Similarly, some men in our study felt that as they had HIV, having multiple concurrent sexual partners, one night stands, and not using condom during sex would attract harsh judgment from their peers and society, thus could no longer occupy this masculine position.

However, we found little or no evidence suggesting that these men cried over the loss of this youthful masculine position, rather they grieved the loss of access to a more valued form of masculinity, the traditional masculine position. The latter masculine position has cultural currency in many African settings. The men who occupy it are seen as exemplifying what manhood is. Thus they are celebrated and marveled at as they have demonstrated maturity and a transition from being a boy into being a responsible man (25, 48).

As such, this culturally informed manner of attaining manhood provides a cultural prescription for young boys and men to measure themselves and their masculinities against (49). To occupy this position, men should perform roles such as finding a stable job, marry and start a family, provide for and sustain it (41, 48). Indeed, most men interviewed in this study aspired to occupy this position, yet the majority of them regrettably felt it was no longer accessible to them as they now had acquired HIV. In general, men closely linked HIV acquisition to inadequacy and loss [of manhood]. This was more apparent in Itumeleng's and Jeff's narratives whom we argue had certainly tapped into some of the aspects of traditional masculinity, but they too viewed the traditional masculine position as unattainable, linking their lack of potency to attain it to their having HIV.

We have noted a sizeable number of men who reconfigured their masculinity after being diagnosed with HIV. We argue that this positional shift in masculinities was in reaction to having been diagnosed with HIV. Through performing more caring roles toward others (50), being concerned about their health and starting and adhering to treatment (48), expressing their needs and emotions and seeking help, these men had clearly departed from the harmful notions of hegemonic masculinity, as these behaviors are clearly in opposition to it (51). It appears that the observed shift was preceded by some critical reflection on the men's part. The possibilities of men changing their behaviors and masculinities have been alluded to by Barker and Ricardo (48). They argued that in Africa, there is evidence that masculinities are changing as ‘the dimensions of the AIDS epidemic in Africa and the devastation of families are forcing some men to question gender norms and attitudes that were once unquestionable’ (p. 44).

Similarly, in our study, the vast majority of men who were able to reconfigure their masculinities into one emphasizing responsibility and caring were likely to be those who had, over the years, mulled over their life circumstances and masculinities, disclosed their status, sought help thus had linkages to care, were on ARVs and adhering. This highlights the critical need to reach out to those men who are living with HIV but may not be sick and thus not eligible for ARVs and ensure that they are linked to support and care. Furthermore, we argue that the men who were unable to reconfigure their masculinities and had remained in the diminished position were particularly vulnerable to negative coping strategies (e.g. drinking alcohol at harmful levels, denial, and other self-destructive behavior) which ultimately were likely to be life shortening.

Interventions to support adjustment to living with HIV need to take into account the impact of HIV on masculinity and the impact of masculinity on coping with a life with HIV and its length. Available gender-transformative interventions in the gender-based violence (GBV) and HIV prevention sectors have sought to change the way men see themselves as men as part of the prevention of GBV and sexually risk behavior. The success of these interventions which are ‘grounded on research-based theoretical models of prevention’ (52) has been documented (53), and the Stepping Stones intervention is an example in South Africa (see 54, 55). The Stepping Stones curriculum comprises a participatory approach that includes critical reflection to encourage safer sexual practices through building more gender-equitable relationships (54). This approach draws on Freire's (56) work in which he argues that through discourse and reflection, people can begin to question themselves and their behavior and pursue alternative ways of being. In working with men for gender-transformation, this approach affords men spaces to reappraise their masculinities and adopt more gender-equitable forms of masculinities (47). Thus, we argue that the same principles of critical reflections could be used with good effect in interventions that seek to promote positive adjustment for men living with HIV in the critical phase after diagnosis and before care, as well as those starting care.

### Strengths and limitations

Most men interviewed in this study were recruited from a HIV clinic in Johannesburg and Soweto. Thus, because they had access to HIV services from the clinics, they may have been different to other men living with HIV in the general population. Notwithstanding, however, in our analysis, in terms of experiences of living with HIV and the behaviors reported, we could not discern any difference between these men (recruited through clinics) and those recruited in the general community in Mthatha.

In this study, we interviewed a man who self-identified as a man who has sex with both men and women. Whilst our analysis revealed no discernable differences in terms of his experiences of living with HIV and constructions of masculinities as compared to those who self-identified as heterosexual, future studies would do well not to mix men who have different sexual orientations as this may introduce gender identity complexities that may be difficult to explicate. We acknowledge that social desirability could be a potential bias in this study as some men may have reported behaviors they felt would be acceptable to be told to the interviewers.

## Conclusion

In this paper we have shown that HIV can have a significant effect on men and their masculinities. Furthermore, we have presented evidence that men's constructions and performance of harmful notions of hegemonic masculinity pre-HIV diagnosis negatively affect their help-seeking behavior and adjustment to living with HIV, post-diagnosis. Interventions with men living with HIV need to provide safe spaces for men to reflect on their life circumstances (including health risk behaviors), and identify significant, meaningful and future-oriented goals and aspirations. These could be the necessary motivations and triggers for men to reconfigure their masculinities and help-seeking behavior. For this change to occur and for men to adopt more gender and progressive masculinities that would enable them to accept, cope, and adjust more easily to living with HIV, the infusion of elements of critical reflection (56) in interventions may be beneficial. Such interventions should critically explore the constructions of social identities and the pressures these place on men. Furthermore, the interventions should vigorously challenge harmful constructions of masculinities, this process may render men amenable to change.
